# Risk factors for severe illness in hospitalized Covid-19 patients at a regional hospital

**DOI:** 10.1371/journal.pone.0237558

**Published:** 2020-08-12

**Authors:** Justin J. Turcotte, Barry R. Meisenberg, James H. MacDonald, Nandakumar Menon, Marcia B. Fowler, Michaline West, Jane Rhule, Sadaf S. Qureshi, Eileen B. MacDonald

**Affiliations:** 1 Department of Orthopedics, Anne Arundel Medical Center, Annapolis, MD, United States of America; 2 Department of Medicine, Anne Arundel Medical Center, Annapolis, MD, United States of America; 3 Anne Arundel Research Institute, Anne Arundel Medical Center, Annapolis, MD, United States of America; National Yang-Ming University, TAIWAN

## Abstract

**Background:**

The Covid-19 pandemic threatens to overwhelm scarce clinical resources. Risk factors for severe illness must be identified to make efficient resource allocations.

**Objective:**

To evaluate risk factors for severe illness.

**Design:**

Retrospective, observational case series.

**Setting:**

Single-institution.

**Participants:**

First 117 consecutive patients hospitalized for Covid-19 from March 1 to April 12, 2020.

**Exposure:**

None.

**Main outcomes and measures:**

Intensive care unit admission or death.

**Results:**

In-hospital mortality was 24.8% and average total length of stay was 11.82 days (95% CI: 10.01 to 13.63 days). 30.8% of patients required intensive care unit admission and 29.1% required mechanical ventilation. Multivariate regression identified the amount of supplemental oxygen required at admission (OR: 1.208, 95% CI: 1.011–1.443, p = .037), sputum production (OR: 6.734, 95% CI: 1.630–27.812, p = .008), insulin dependent diabetes mellitus (OR: 11.873, 95% CI: 2.218–63.555, p = .004) and chronic kidney disease (OR: 4.793, 95% CI: 1.528–15.037, p = .007) as significant risk factors for intensive care unit admission or death. Of the 48 patients who were admitted to the intensive care unit or died, this occurred within 3 days of arrival in 42%, within 6 days in 71%, and within 9 days in 88% of patients.

**Conclusions:**

At our regional medical center, patients with Covid-19 had an average length of stay just under 12 days, required ICU care in 31% of cases, and had a 25% mortality rate. Patients with increased sputum production and higher supplemental oxygen requirements at admission, and insulin dependent diabetes or chronic kidney disease may be at increased risk for severe illness. A model for predicting intensive care unit admission or death with excellent discrimination was created that may aid in treatment decisions and resource allocation. Early identification of patients at increased risk for severe illness may lead to improved outcomes in patients hospitalized with Covid-19.

## Introduction

In December 2019, China reported deaths attributed to the flu-like syndrome Covid-19 [1]. By July 2020 there have been over 14M patients diagnosed with Covid-19 and 600,000 deaths worldwide, with over 3.9M diagnosed and 142,000 deaths in the United States [2].

The primary objective of this analysis was to identify risk factors for severe illness, defined as ICU admission or death. A secondary objective was to use these risk factors to develop a predictive model that can be applied in practice for external validation. Although some data has described predictors of critical care needs [3], this has not been validated in different populations and at different medical centers. Others have published on predictors of critical illness. Liang et al. described a group of variables that were independent factors predicting for a more serious illness: chest radiographic abnormality, age, hemoptysis, dyspnea, unconsciousness, number of comorbidities, cancer history, neutrophil-to-lymphocyte ratio, lactate dehydrogenase, and direct bilirubin [3]. Garg et al. concluded that underlying medical comorbidities, older age, diabetes, obesity, and male sex identified biological vulnerabilities for more severe Covid-19 outcomes [4]. Zhou et al. showed increasing odds of in-hospital death associated with older age, higher Sequential Organ Failure Assessment (SOFA) score, and d-dimer greater than 1 μg/mL on admission [5]. In the largest cohort study to date of 17 million adults in England, Williamson et al. demonstrated Covid-19 death was associated with male gender, older age, deprivation, diabetes, severe asthma, and multiple other comorbidities [6]. In addition, multiple studies have proposed models for predicting severe illness based upon demographic, comorbidity and radiologic data. However, substantial variability in the predictive accuracy and methodological quality of these studies exists, thus warranting further investigation into the utility of these tools [7].

We sought to identify factors at presentation that are associated with ICU admission or death and clinical findings during hospitalization that may indicate sustainable improvement that would allow for transition to a lower level of care. Early identification of patients at risk for developing severe illness may aid in resource allocation decisions and improve outcomes in patients with Covid-19.

## Methods

This study was reviewed by the institutional clinical research committee (the Anne Arundel Research Institute Clinical Research Committee) and deemed to be exempt research (study # 1590551–2). The need for patient consent was waived as a category 4 exemption of secondary research for which consent is not required. All information was recorded so that subjects could not be identified directly or through linked identifiers; subjects were not re-identified or contacted.

### Setting

Anne Arundel Medical Center is a 350-bed acute care hospital with over 25,000 admissions, and 95,000 Emergency Department (ED) visits annually.

### Data collection

A retrospective, chart review of the first 117 consecutive patients hospitalized for Covid-19 from March 1 to April 12, 2020 was conducted. All adult patients > 18 years were confirmed Covid-19 positive by real-time reverse-transcription polymerase-chain-reaction (RT-PCR) assay. All patients included in the study stayed at least one midnight in the hospital. Patients discharged on the day of arrival from the ED or observation unit, deceased on the day of arrival, or not confirmed Covid-19 positive by RT-PCR assay were excluded.

Data definitions were standardized by board certified internal medicine physicians prior to collection. The electronic medical record was manually abstracted to identify patient demographics, objective and subjective presentation at arrival, comorbidities present on arrival, lab values on day zero or one of hospitalization, oxygen saturations and requirements for supplemental oxygen, level of care required, mechanical ventilation requirements, and hospital discharge disposition. For each day of hospitalization, mechanical ventilation or supplemental oxygen requirements, the maximum liters per minute of supplemental oxygen required for non-ventilated patients, and the lowest oxygen saturation of the patient over each 24-hour period was recorded. Total length of stay (TLOS) was defined as the day count from arrival in ED to day of discharge or death in order to eliminate variability in time from ED arrival to inpatient status. The primary endpoint was ICU admission or in-hospital death. ICU admission was considered for patients that had a) maximal or near maximal high-flow nasal cannula therapy (>30L flow or 90% FiO2); b) rapidly declining status (significantly increased support over 6–8 hr. period or impending need for intubation); c) additional end organ dysfunction (for example hypotension/cardiomyopathy, altered mental status, etc.) or d) other traditional criteria for ICU admission. The final decision for ICU transfer was made by an attending intensivist in consultation with patients and families depending on patient-specific factors including overall prognosis and functional status prior to admission. All deaths during the study period were due to Covid-19. While the exact cause of death was often multifactorial, all were related to the Covid-19 disease process.

### Data analysis

Descriptive statistics were performed for the entire population. The population was then stratified by whether a patient received ICU care and/or died in the hospital. Univariate comparisons using 2-sided independent sample t-tests for continuous, and chi-square or Fisher’s Exact test when indicated for categorical variables were performed. For multivariate assessment of risk factors and predictive model creation, all variables with a p value of < .2 on univariate analysis were entered into a stepwise forward conditional multivariate logistic regression model with an endpoint of ICU admission or death. Adjusted odds ratios controlling for all other confounding variables included in the final model were evaluated to assess the association between each risk factor and severe illness. Lab values were not included in the model to reduce bias, as tests were not uniformly performed on all admitted patients in the early phases of the Covid-19 pandemic in our region. Additionally, six patients that were placed on a ventilator at the time of arrival were excluded from the multivariate analysis. The Hosmer and Lemeshow test was used to assess model fit, and discrimination was assessed by the area under the curve (AUC) of the receiver operating characteristic (ROC) curve when the model was applied to the patient population. A series ROC curves were then constructed to evaluate the AUC of the individual risk factors identified. Upon completion of the multivariate evaluation of the primary endpoint (ICU admission or death), enter method logistic regression using the risk factors identified in the model was performed to evaluate the endpoints of ICU admission and death separately, and sensitivity analysis was performed to assess the impact of including significant lab values from univariate analysis. Cox proportional hazards regression was then performed to evaluate the relationship between the risk factors and survival time to ICU admission or death. All statistical analysis was performed using SPSS version 26 (IBM, Armonk, NY).

## Results

One hundred seventeen patients were included in this study. Patients had an average age of 65.7 ± 15.5 yrs. 55.3% of the population was white while 28.2% was black or African American. The most common findings at presentation were cough (82.1%), shortness of breath (78.6%), and fatigue (67.5%). The average lowest oxygen saturation on the day of arrival was 90.9 ± 15.1%; 5.1% of patients required a ventilator emergently and 37.6% of patients required supplemental oxygen. The average maximum supplemental oxygen requirement for patients requiring it on the day of arrival was 4.7 ± 6.3 L/min. Chest computed tomography was performed in 110 patients, and ground glass opacities were identified in 58.1% of these scans. The most common clinical comorbidities at presentation were hypertension (65.8%), lung disease (29.1%), and non-insulin dependent diabetes mellitus (NIDDM, 26.5%) (Table 1). Of the 117 patients in this study, 15 (12.8%) remained admitted at the time of analysis. Their average total length of stay (ATLOS; 31.3 ± 7.7 days) was calculated as of the study date, and 11 (73.3%) were admitted to the ICU. These patients were included in all analyses.

**Table 1:** Characteristics of patients requiring ICU care or discharged deceased.

Patient Characteristic (N No ICU/Death, N ICU/Death for measures not available for total sample)	All Patients N = 117	No ICU or Death N = 69	Admitted to ICU or Discharged Deceased N = 48	P Value
Demographics				
Age (yrs)–Avg ± SD	65.7 ± 15.5	62.6 ± 16.9	70.2 ± 12.1	**.009**
BMI (kg/m^2^)–Avg ± SD	31.4 ± 9.1	31.1 ± 9.5	32.0 ± 8.4	.606
Female Gender–N (%)	55 (47.0)	33 (47.8)	22 (45.8)	.832
Non-White Race/Ethnicity–N (%) (N = 65, N = 40)**	47 (44.7)	29 (44.6)	18 (45.0)	.969
Clinical Presentation				
Objective Findings				
Respiratory Rate (breaths/min)–Avg ± SD	21.3 ± 5.6	21.06 ± 5.21	21.65 ± 6.04	.575
Heart Rate (beats/min)–Avg ± SD	92.1 ± 19.2	93.55 ± 17.18	90.06 ± 21.87	.337
Temperature (°F) –Avg ± SD	99.2 ± 2.1	99.57 ± 1.47	98.60 ± 2.73	**.015**
Fever ≥ 99.5° F–N (%)	49 (41.9)	37 (53.6)	12 (25.0)	**.002**
ST Segment Elevation–N (%)	8 (6.8)	6 (8.7)	2 (4.2)	.468*
O2 Saturation–Avg ± SD	90.9 ± 15.9	91.75 ± 16.44	89.75 ± 15.10	.504
Required Ventilator–N (%)	6 (5.1)	1 (1.4)	5 (10.4)	.042*
Required Supplemental O2 –N (%)	44 (37.6)	22 (31.9)	22 (46.8)	.104
Amount Supplemental O2 Required (L/min)–Avg ± SD	4.7 ± 6.3	2.83 ± 3.64	6.77 ± 8.04	**.028**
Ground Glass on CT Scan–N (%) (N = 64, N = 46)	68 (61.8)	39 (60.9)	29 (63.0)	.823
Subjective Findings				
Myalgia–N (%)	38 (32.5)	30 (43.5)	8 (16.7)	**.002**
Fatigue–N (%)	79 (67.5)	52 (75.4)	27 (56.3)	**.030**
Headache–N (%)	12 (10.3)	8 (11.6)	4 (8.3)	.759*
Rhinorrhea–N (%)	15 (12.8)	9 (13.0)	6 (12.5)	.931
Shortness of Breath–N (%)	92 (78.6)	56 (81.2)	36 (75.0)	.424
Chest Pain–N (%)	44 (37.6)	28 (40.6)	16 (33.3)	.426
Cough–N (%)	96 (82.1)	60 (87.0)	36 (75.0)	.097
Sputum–N (%)	13 (11.1)	4 (5.8)	9 (18.8)	**.028**
Hemoptysis–N (%)	3 (2.6)	1 (1.4)	2 (4.2)	.567
Nausea or Vomiting–N (%)	23 (19.7)	19 (27.5)	4 (8.3)	**.010**
Diarrhea–N (%)	36 (30.8)	23 (33.3)	13 (27.1)	.471
Total Number of Symptoms–Avg ± SD	3.9 ± 1.9	4.29 ± 1.78	3.40 ± 2.06	**.014**
Days Since Onset–Avg ± SD	8.5 ± 9.1	8.63 ± 7.18	8.38 ± 11.46	.890
Comorbidities and Risk Factors				
Non-Insulin Dependent Diabetes Mellitus	31 (26.5)	16 (23.2)	15 (31.3)	.331
Insulin Dependent Diabetes Mellitus	15 (12.8)	2 (2.9)	13 (27.1)	**< .001**
Obstructive Sleep Apnea	11 (9.4)	3 (4.3)	8 (16.7)	.049*
Any Lung Disease (COPD or Asthma)	34 (29.1)	18 (26.1)	14 (29.2)	.713
Immunosuppressed	15 (12.8)	7 (10.1)	8 (16.7)	.299
Long Term Steroid Use	10 (8.5)	7 (10.1)	3 (6.3)	.523*
Atrial Fibrillation	15 (12.8)	4 (5.8)	11 (22.9)	**.006**
Congestive Heart Failure	22 (18.8)	9 (13.0)	13 (27.1)	.056
Coronary Artery Disease	26 (22.2)	12 (17.4)	14 (29.2)	.132
Chronic Kidney Disease	27 (23.1)	7 (10.1)	20 (41.7)	**< .001**
On Dialysis	3 (2.6)	1 (1.4)	2 (4.2)	.567*
Gastroesophageal Reflux Disease	21 (17.9)	13 (18.8)	8 (16.7)	.763
Hypertension	77 (65.8)	42 (60.9)	35 (72.9)	.177
History of Smoking	43 (36.8)	25 (36.2)	18 (37.5)	.889
Current Smoker	5 (5.1)	5 (7.2)	1 (2.1)	.211*
Consumes Alcohol	39 (33.3)	22 (31.9)	17 (35.4)	.690
Number of Comorbidities/Risk Factors–Avg ± SD	3.6 ± 2.3	3.04 ± 2.06	4.46 ± 2.34	**.001**

Significant P Values < .05 in bold

* Fisher’s Exact Test, if not otherwise denoted independent samples t-test used for continuous and chi-square test used for categorical variables

**Presented as a percent of patients reporting race or ethnicity, excludes 12 patients unknown or did not reply

BMI–body mass index

O2 –oxygen

CT–chest computed tomography

In-hospital mortality was 24.8% and overall ATLOS was 11.8 days (95% CI: 10.01 to 13.63 days). 30.8% of patients required ICU admission and 29.1% required mechanical ventilation. The average day of ICU admission was 3.4 (95% CI: 1.98 to 4.83). ATLOS for ICU patients was 18.4 days (95% CI 14.35 to 22.38 days) and 47.2% of ICU patients expired in the hospital. 69 patients (58.9%) survived to discharge and did not require ICU care. The ATLOS for these patients was 9.26 days (95% CI: 7.45 to 11.07 days) (Table 2).

**Table 2:** Patient outcomes.

Outcome Measure	Result
All Patients–N (%)	117 (100.0)
Discharge Disposition–N (%)	
In-hospital Death	29 (24.8)
Hospice	2 (1.7)
Transfer to Other Acute Care Facility	1 (0.9)
SNF	14 (12.0)
Home with Home Health	4 (3.4)
Home Self Care	52 (44.4)
Still Admitted*	15 (12.8)
Total Length of Stay Days–Avg ± SD	11.82 ± 9.86
Total Length of Stay Days– 95% CI	10.01–13.63
Total Length of Stay Days–Median	9.00
Admitted to ICU–N (%)	36 (30.8)
Day of ICU Admission–Avg ± SD	3.41 ± 4.28
Day of ICU Admission– 95% CI	1.98–4.83
ICU Length of Stay Days–Avg ± SD	14.86 ± 11.35
Total Length of Stay Days–Avg ± SD	18.36 ± 11.86
Total Length of Stay Days– 95% CI	14.35–22.38
Total Length of Stay Days–Median	20.00
In-hospital Death	17 (47.2)
Required Ventilator–N (%)	34 (29.1)
Admitted to ICU–N (%)	33 (97.1)
Day of Ventilator Start—Avg ± SD	3.41 ± 4.01
Day of Ventilator Start– 95% CI	2.01–4.81
Ventilator Days–Avg ± SD	11.74 ± 8.17
Ventilator Days– 95% CI	8.88–14.59
Ventilator Days—Median	9.00
In-hospital Death–N (%)	15 (44.1)
Patients Alive and Not Requiring ICU Admission–N (%)	69 (58.9)
Total Length of Stay Days–Avg ± SD	9.26 ± 7.53
Total Length of Stay Days– 95% CI	7.45–11.07
Total Length of Stay Days–Median	8.00
All Patients Alive–N (%)	88 (75.2%)
Total Length of Stay Days–Avg ± SD	12.68 ± 10.47
Total Length of Stay Days– 95% CI	10.46–14.90
Total Length of Stay Days–Median	9.00
All Patients Discharged Deceased–N (%)	29 (24.8%)
Total Length of Stay Days–Avg ± SD	9.21 ± 7.28
Total Length of Stay Days– 95% CI	6.44–11.98
Total Length of Stay Days–Median	7.00

*15 patients still admitted as of date of analysis. ATLOS Days = 31.27 ± 7.69

SNF- skilled nursing facility

ICU–intensive care unit

In comparison to patients living and not requiring ICU care, those who died or went to the ICU were older (70.2 vs. 62.6 yrs, p = .009), had lower maximum temperature (98.6 vs. 99.6°F, p = .015), lower rates of fever (25.0% vs. 53.6%, p = .002), higher rates of ventilator use (10.4% vs. 1.4%, p = .042), and required more supplemental oxygen (6.77 vs. 2.83 L/min, p = .028) on the day of hospital arrival. On subjective evaluation, patients who required ICU or died had lower rates of myalgia (56.3% vs. 75.4%, p = .002), fatigue (16.7% vs. 43.5%, p = .002), and nausea or vomiting (8.3% vs. 27.5%, p = 010), were more likely to complain of increased sputum production (18.8% vs. 5.8%, p = .028), and had fewer total symptoms (3.4 vs. 4.29, p = .014). Patients dying or going to the ICU had more total comorbidities or risk factors (4.5 vs. 3.0, p = .001) and displayed higher rates of insulin-dependent diabetes mellitus (IDDM, 27.1% vs. 2.9%, p < .001), obstructive sleep apnea (16.7% vs. 4.3%, p = .049), atrial fibrillation (22.9% vs. 5.8%, p = .006), and chronic kidney disease (CKD) (41.7% vs. 10.1%, p < .001). On hospital day zero or one, they had higher WBC count (9.0 ± 4.4 vs. 7.1 ± 3.3, p = .014) and higher D-Dimer levels (3.2 ± 4.5 vs 1.5 ± 1.8, p = .034) (Table 3).

**Table 3:** Lab values on day zero or one of hospitalization.

Lab Value (N No ICU/Death, N ICU/Death for measures not available for total sample)	All Patients N = 117	No ICU or Death N = 69	Admitted to ICU or Discharged Deceased N = 48	P Value
WBC (10^3^/μL) (N = 68, N = 48)	7.84 ± 3.87	7.06 ± 3.25	8.95 ± 4.40	**.014**
Lymphocytes (10^3^/μL) (N = 65, N = 46)	1.53 ± 3.03	1.50 ± 2.60	1.57 ± 3.58	.909
Hemoglobin (g/dL) (N = 68, N = 48)	12.39 ± 2.31	12.64 ± 2.16	12.04 ± 2.48	.178
Platelets (10^3^/μL) (N = 67, N = 47)	226.87 ± 100.47	232.08 ± 97.86	219.46 ± 104.70	.517
ALT (IU/L) (N = 64, N = 46)	48.57 ± 49.80	53.78 ± 60.00	41.33 ± 29.58	.197
AST (IU/L) (N = 63, N = 46)	59.08 ± 51.97	56.65 ± 55.59	62.41 ± 46.97	.560
PT (sec) (N = 14, N = 12)	16.00 ± 6.16	14.91 ± 3.84	17.26 ± 8.09	.343
PTT (sec) (N = 10, N = 16)	36.06 ± 11.50	34.80 ± 9.47	36.84 ± 12.84	.668
FDP (μg/mL) (N = 9, N = 15)	685.00 ± 215.99	630.33 ± 73.41	717.80 ± 265.49	.246
D-Dimer (μg/mL FEU) (N = 52, N = 37)	2.23 ± 3.29	1.52 ± 1.82	3.23 ± 4.48	**.034**
CK (IU/L) (N = 18, N = 17)	347.94 ± 418.37	381.78 ± 495.52	312.12 ± 329.25	.630
LDH (IU/L) (N = 45, N = 34)	379.08 ± 203.41	360.77 ± 206.90	403.30 ± 199.14	.361
ESR (mm/hr) (N = 29, N = 25)	85.83 ± 35.82	82.17 ± 33.05	90.08 ± 39.05	.424
CRP (mg/dL) (N = 50, N = 35)	9.76 ± 6.27	8.67 ± 6.19	11.32 ± 6.14	.056
Procalcitonin (ng/mL) (N = 49, N = 35)	0.59 ± 1.49	0.35 ± 0.96	0.91 ± 1.99	.094
Ferritin (ng/mL) (N = 53, N = 37)	1,173.78 ± 1,623.20	1408.44 ± 2009.07	837.65 ± 697.34	.101
Troponin ≤ 0.06 –N (%) (N = 68, N = 45)	67 (59.3)	42 (61.8)	25 (55.6)	.511
Troponin 0.061–2.0 –N (%)	11 (9.7)	4 (5.9)	7 (15.6)	.111*
Troponin > 2.0 –N (%)	1 (0.9)	1 (1.5)	0 (0.0)	1.000*

Significant P Values < .05 in bold

* Fisher’s Exact Test, if not otherwise denoted independent samples t-test used for continuous and chi-square test used for categorical variables

WBC–White Blood Cells

ALT—Alanine Aminotransferase

AST—Aspartate Aminotransferase

PT—Prothrombin Time

PTT—Partial Thromboplastin Time

FDP–Fibrin Degradation Products

FEU–Fibrinogen Equivalent Units

CK–Creatinine Kinase

LDH—Lactate Dehydrogenase

ESR—Erythrocyte Sedimentation Rate

CRP—C Reactive Protein

**Fig 1:**
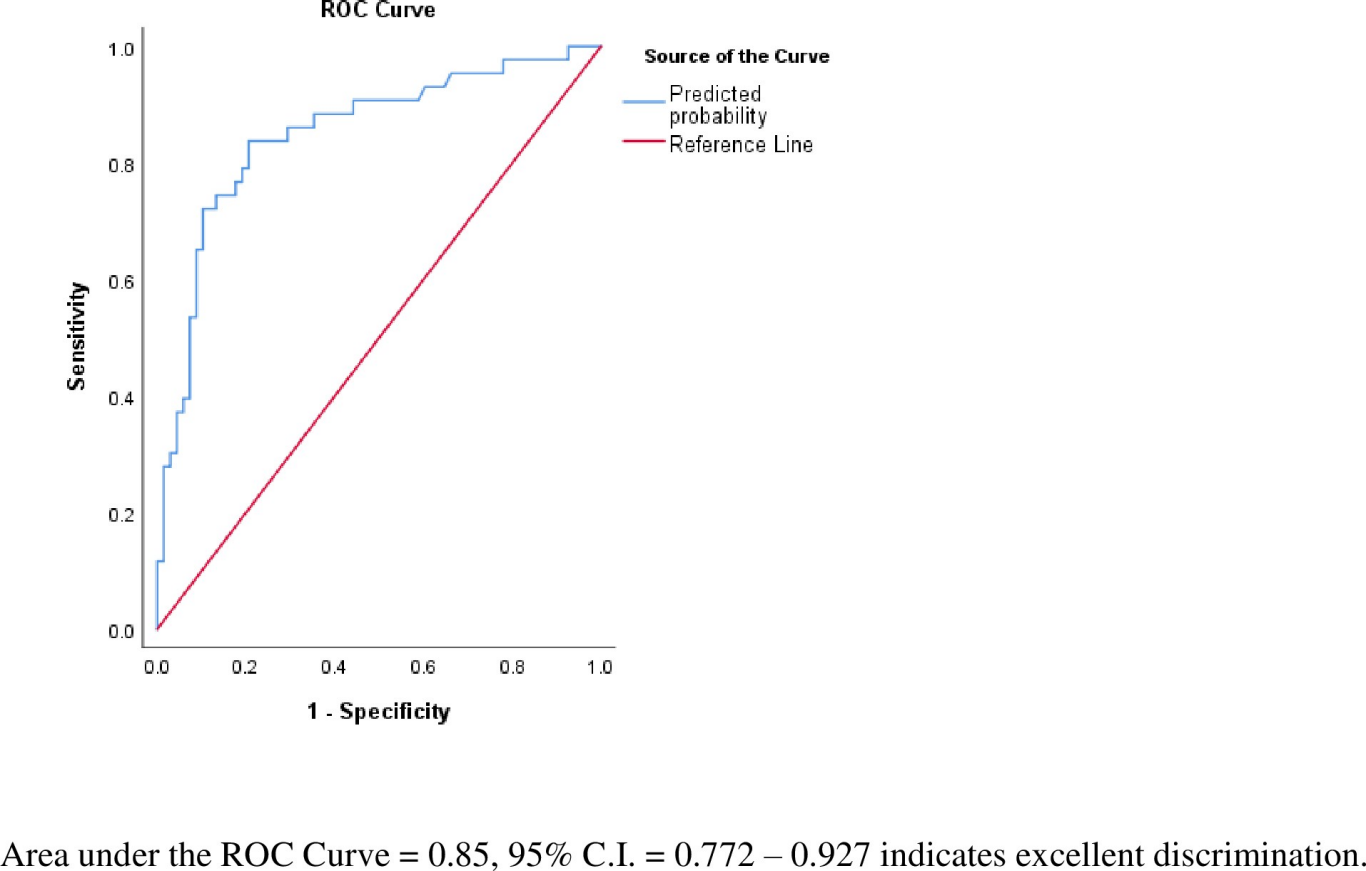
Receiver operating characteristics curve of model predictive value.

Multivariate logistic regression identified the amount of supplemental oxygen required at admission, subjective complaint of increased sputum, and comorbidities of IDDM and CKD as significant risk factors for ICU admission or death. Using these variables plus the non-statistically significant variable of patient temperature at admission, ICU admission or death could be predicted with an area under the ROC curve of 0.85 (95% CI: 0.772 to 0.927), indicating excellent discriminatory ability [8] (Fig 1). At the optimal cutoff point of 26.3% probability of ICU admission or death, the model had a sensitivity of 83.7% and specificity of 79.4%, and demonstrated adequate fit using the Hosmer and Lemeshow test (χ^2^ = 5.306, p = .724). Through the 7 step forward conditional regression process, the following variables were eliminated: age, whether supplemental oxygen was required at admission, myalgia, fatigue, cough, nausea or vomiting, obstructive sleep apnea, atrial fibrillation, coronary artery disease, hypertension, the number of risk factors present at admission, and the number of symptoms at admission. Details of the variables excluded from the model and the step summary of model development are included as supporting information S1 and S2 Tables. The variables included in this final model, along with adjusted odds ratios and 95% confidence intervals are presented in Table 4. The model equation follows:

$$p=\frac{1}{1+e^{-(22.316{-.242}_{temp.}+.189_{O2L}+1.907_{sputum}+2.474_{IDDM}+1.567_{CKD})}}$$

**Table 4:** Multivariate logistic regression analysis: Presentation predictors of ICU admission or death.

Independent Variable	B				95% C.I. for Odds Ratio	
		S.E.	Wald	Odds Ratio	Lower	Upper	P Value
Temperature at Admission (°F)	-.242	.130	3.489	0.785	0.609	1.012	.062
Supplemental O_2_ at Admission (L/min)	.189	.091	4.339	1.208	1.011	1.443	**.037**
Sputum Production	1.907	.724	6.946	6.734	1.630	27.812	**.008**
Insulin Dependent Diabetes Mellitus	2.474	.856	8.356	11.873	2.218	63.555	**.004**
Chronic Kidney Disease	1.567	.583	7.218	4.793	1.528	15.037	**.007**
Constant	22.316	12.834	3.023	N/A	N/A	N/A	.082

Significant P Values < .05 in bold

S.E.–standard error

O_2_—oxygen

**Fig 2:**
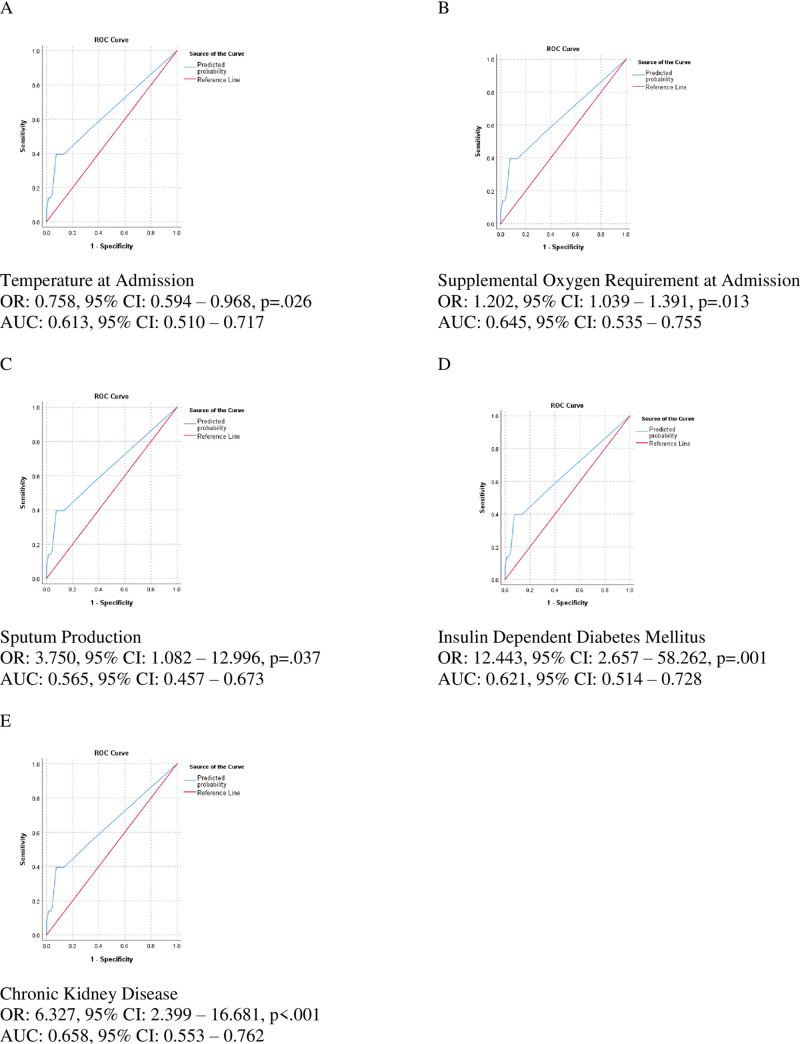
Univariate logistic regression results and ROC curve analyses of individual predictors of ICU admission or death.

**Fig 3:**
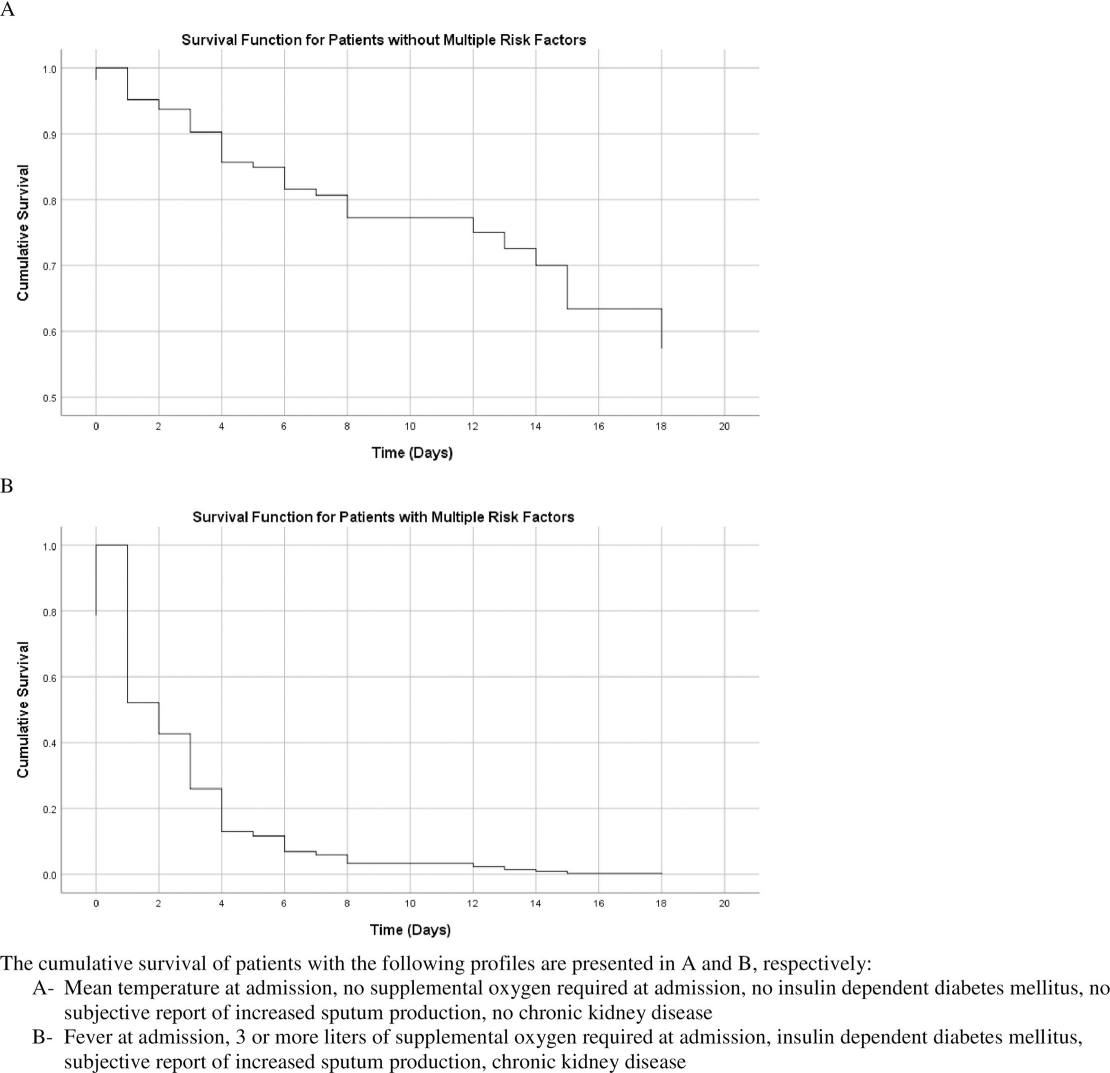
Survival curves of time to ICU admission or death by patient risk profile.

To further evaluate the value of these five variables in predicting adverse outcomes, multivariate logistic regression using the enter method was performed on the endpoints of death and ICU admission individually, and ROC analysis was performed to assess discrimination. When applied to the death endpoint, the model yielded an AUC of 0.88 (95% CI: 0.812–0.956), while evaluation of ICU admission yielded an AUC of 0.74 (95% CI: 0.628–0.849). The outputs of each regression model are presented in S3 and S4 Tables. Univariate logistic regression and ROC curve analyses were performed to assess each independent variable’s AUC in relation to the primary endpoint of ICU admission or death. Each of the five independent variables was significantly associated with increased risk of ICU admission or death, with temperature at admission demonstrating an inverse relationship. AUCs of the ROC curves ranged from 0.57 for subjective complaint of increased sputum production to 0.66 for chronic kidney disease. The odds ratios, 95% confidence intervals from regression, and AUCs of each independent variable are presented in Fig 2. Sensitivity analysis was performed to evaluate whether addition of the significant lab values on univariate analysis, WBC count and D-Dimer, impacted model performance. Neither WBC count nor D-Dimer were significant when added to the model (S5 Table), and no significant improvement in AUC was observed when applying the model to the ICU admission or death endpoint (AUC = 0.85, 95% CI: 0.751–0.940, p = .435). Cox proportional hazard regression using the enter method and the five independent variables from the previous model was performed to assess survival time to ICU admission or death. Increased supplemental oxygen requirements at admission and IDDM (both p = .001) were found to be statistically significant risk factors for decrease survival time, while temperature at admission, subjective complaint of increased sputum production, and chronic kidney disease did not reach statistical significance. The results of the Cox regression model are presented in S6 Table, and survival functions for patients with varying levels of risk factors are presented in Fig 3.

**Fig 4:**
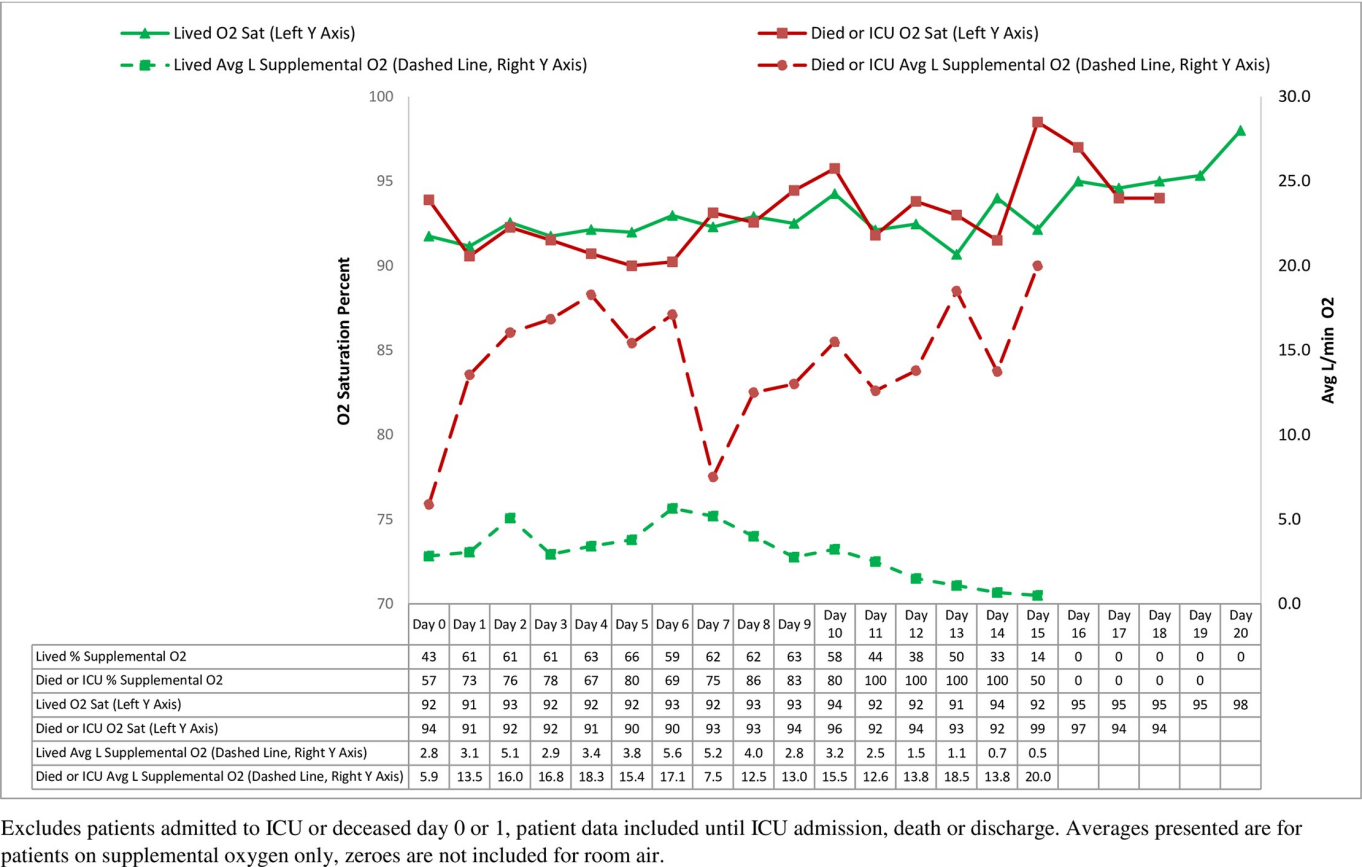
Oxygen saturation and supplemental oxygen by day.

Of the 48 patients who were admitted to the ICU or died, this occurred within 3 days of arrival in 42%, within 6 days of arrival in 71%, and within 9 days of arrival in 88% of patients. Of the patients who lived and did not go to the ICU, 65% were discharged in 9 days or less. Trends in oxygen saturation and supplemental oxygen utilization rates by hospital day prior to either ICU admission or death are presented in Fig 4. Throughout the course of care, all patients maintained similar levels of oxygen saturation. While no statistically significant differences by day exist, patients who were admitted to the ICU or died demonstrated a trend toward higher average levels of supplemental oxygen requirement throughout their hospital course (min.-max.: 5.9–20.0 L/min). Those who lived and did not go to the ICU appeared to require less supplemental oxygen (min.-max.: 0.5–5.6 L/min) and demonstrated a consistent decline in average liters/minute required after hospital day seven.

## Discussion

In patients hospitalized for Covid-19, we identified notable differences in demographics, comorbidities and clinical presentations between patients who experienced severe illness and those who did not. This study is novel in that it presents risk factors for ICU admission or death and proposes a model for predicting which patients will experience these outcomes. Finally, we examined the time course of the disease and identified oxygenation patterns that may aid clinicians in developing guidelines for discharge to lower levels of care. Early identification of patients at risk for severe illness and determining the optimal length of stay that strikes the delicate balance between freeing acute care resources, ensuring the safety of patient transition to lower level care, and minimizing opportunity for further viral transmission, remains a nuanced patient specific decision that carries immense public health implications.

### Characteristics of patients with ICU admission or death

Rates of ICU admission, ICU mortality, and overall mortality at our institution were similar to previously reported rates of 26% to 32% [5, 9–11] 39 to 72% [5, 9, 10, 12, 13] and 10 to 21% respectively [14, 15]. ICU admission rates and mortality are likely affected by the goals of care discussions with ED, hospital or ICU medical staff.

In our patient population, significantly higher rates of IDDM, OSA, atrial fibrillation, chronic kidney disease, and cumulative number of comorbidities were observed in patients with ICU admission or death. On multivariate analysis, only IDDM and chronic kidney disease remained significantly associated with increased risk of these endpoints. Previously identified risk factors for severe illness, complications, and death in Covid-19 patients include increased age and comorbidities [5, 9, 11, 13, 16–18]. A closer examination of diabetes specifically reveals similarities between our findings and those of Wang et al., who found 22.2% of patients requiring ICU care had diabetes, compared to 5.9% of non-ICU patients (p = .009) [11]. Although this study did not delineate between NIDDM and IDDM, the finding is similar to our IDDM rate of 27.1% in patients with ICU admission or death compared to 2.9% in non-ICU, alive patients. Similarly, Guan et al. found that 26.9% of patients experiencing ICU admission, mechanical ventilation or death were diabetic compared to 6.1% of patients not reaching this composite outcome [17]. Richardson et al. found that of patients who died, those with diabetes were more likely to have received mechanical ventilation or ICU care compared to those without diabetes, and that the percentage of patients who developed acute kidney injury was increased in the diabetic subgroup [14]. In all the case series it is not known if diabetes is a surrogate marker for microvascular disease, kidney disease (even if subclinical) or carries with it certain medications which are themselves risk factors for more serious infection or immune response. Pre-admission medication use was not examined as part of this analysis. Based on our finding that both IDDM and chronic kidney disease were predictors of ICU admission or death, and the alignment with other case series, further investigation into the mechanism by which Covid-19 impacts renal function is warranted to determine if any of these pre-dispositions are preventable.

At initial presentation, patients who were admitted to the ICU or died had statistically significantly lower temperatures, and lower rates of myalgia, fatigue, and nausea or vomiting. They required higher volumes of supplemental oxygen, had higher rates of sputum production, but a lower overall number of symptoms. On multivariate analysis, subjective report of increased sputum production and amount of supplemental oxygen required at admission were significantly associated with increased risk of ICU admission or death. These results demonstrate the variability in clinical presentation, and difficulty in assessing eventual patient decline based on symptom burden. The inverse relationship between traditional flu-like symptoms [19] (fever, myalgia, fatigue, and nausea or vomiting) and ICU admission or death is notable, as these commonly used screening measures may be of limited prognostic value. Our results are largely in alignment with the following previously reported rates of symptoms at presentation: cough (59–82%), fatigue (44–70%), myalgia (11–35%), and less common symptoms (<10%)–headache, rhinorrhea, sore throat, hemoptysis, vomiting [9, 11, 12, 17, 18, 20, 21]. In comparison to other studies, we observed lower rates of sputum production and fever, and higher rates of shortness of breath and diarrhea [9, 11, 12, 22].

In our study, early lab values were of limited utility in predicting patient outcome and call into question the ongoing use of these lab parameters as either monitors of disease or as triggers for use of specific treatments. While we observed statistically significantly higher WBC counts and D-Dimer levels in patients with severe illness, neither lab value was associated with increased risk of ICU admission or death when incorporated into sensitivity analysis of the multivariate model. In contrast, Wang et al. found multiple lab values to be elevated at admission for patients experiencing ICU/ventilator care or death, including higher white blood cell and neutrophil counts, as well as higher levels of D-dimer, creatine kinase, and creatine [23]. Goyal et al. observed that patients requiring mechanical ventilation were more likely to have elevated liver function values and inflammatory markers including ferritin, D-dimer, C-reactive protein, and procalcitonin than those who did not receive invasive ventilation [15]. Based on the variability of these findings we suggest further assessment using larger multi-center databases is required before a conclusion can be reached regarding the of the utility of early lab values in predicting patient outcomes.

Other risk factors that have previously been associated with severe illness but were not found to be significant in our multivariate analysis include hypertension, COPD, and smoking status. Gao et al. observed that after adjustment for confounders, patients with hypertension had a two-fold increase in the relative risk of mortality as compared with patients without hypertension (4.0% vs. 1.1%, HR: 2.12, 95% CI: 1.17–3.82, p = 0.013) [24]. Wu et al. found a case fatality rate (CFR) of 6.3% in patients with COPD, in comparison to an overall CFR of 2.3% [18], and a meta-analysis of seven studies concluded that patients with COPD are at a five-fold higher risk of developing severe Covid-19 infection [25]. Finally, a systematic review and meta-analysis from 15 studies found that COPD patients were at a higher risk of more severe disease (63%, compared to patients without COPD, 33.4% [calculated RR, 1.88, 95% CI: 1.4–2.4]), and that COPD was associated with higher mortality rates of 60%, in comparison to a crude overall mortality rate of 7.4%. In addition, current smokers had a mortality rate of 38.5% and were 1.45 times more likely (95% CI: 1.03–2.04) to have severe complications compared to former and never smokers [26]. While we did not observe significant association between hypertension, COPD, or smoking status and severe illness or death in our patient population, important confounding factors such as length of smoking history, comorbidity severity, and pharmacologic treatment for these conditions were not incorporated in our study.

### Predictive model

The development of a model for predicting ICU admission or death at the time of admission is a novel aspect of our work. Using five readily available data points, a model with excellent discrimination (AUC = 0.85, 95% C.I. = 0.772–0.927) was constructed. While we have demonstrated the feasibility of predictive modeling in this population, further investigation of the model’s utility is required. As our study relied on a relatively small cohort of 117 patients, external validation of the model presented must be conducted before it is considered for use in practice. Additionally, our model demonstrates the complex interactions between risk factors that influence outcomes in this population, as the overall model AUC of 0.85 was significantly higher than that of any individual independent variable (AUC range 0.57 to 0.66). Beyond evaluating the discriminatory ability of the model quantitatively, the difference in survival curves between patients with and without the significant risk factors identified provides a graphical depiction of the negative influence the presence of these risk factors has on prognosis (Fig 3). Multiple alternative approaches to predictive modeling of diagnosis of Covid-19, risk factors for hospital admission, and prognosis have been proposed [7, 22, 27]. Alternative prognostic modeling techniques that have been presented included nomograms, decision trees and chest CT based scoring. These models have produced C-index results (a measure of concordance that is a generalization of AUC that can account for censored data) [28] ranging from 0.90–0.98 for mortality [29, 30], 0.92 to 0.96 for length of stay over 10 days [31], and 0.85–0.95 for severe or critical condition [7, 32, 33]. It is essential that these models be critically assessed, as the emergence of unreliable and inaccurate predictions could cause more harm than benefit [7].

### Clinical course

Identifying the earliest time for safe discharge remains a challenge in the management of patients with Covid-19. In a subset of 33 of 138 patients with a known complete clinical course, Wang et al. noted that throughout the course of hospitalization, most patients had lymphopenia, and non-survivors developed more severe lymphopenia over time; white blood cell counts and neutrophil counts were higher in non-survivors than in survivors; D-dimer was higher in non-survivors than in survivors; and as the disease progressed and clinical status deteriorated, the levels of blood urea and creatinine progressively increased before death [11]. To supplement this description of fluctuations in biomarkers, we examined the dynamic nature of oxygen saturation and supplemental oxygen requirements throughout their hospitalization. Based upon the following observations, we suggest that supplemental oxygen requirements, especially at levels beyond those delivered by low-flow nasal cannula remain an important clinical sign for evaluating disease severity. In our study, patients eventually requiring ICU care or dying a) had higher rates of supplemental oxygen utilization at admission (although not statistically significant), b) required statistically significantly higher volumes of supplemental oxygen at admission and c) demonstrated a trend toward higher average volumes of supplemental oxygen throughout their hospital course prior to ICU admission or death. In examining this time-based trend, hospital day seven appeared to be an important inflection point where patients not requiring ICU care or dying displayed decreased supplemental oxygen requirements. Of critical importance, these differences in supplemental oxygen requirements by day were not statistically significant, and require further investigation using larger sample sizes before they may be considered as indicators for hospital discharge. Further, the hospital day of a patient's illness is dependent upon the time that the patient chooses to present to the hospital.

As a single-institution retrospective case series, this study has notable limitations and may not be representative of the broader population of patients hospitalized with Covid-19. First, the decision to admit patients was based upon the clinical judgment of ED physicians, and exposes this study to selection bias. Similarly, our composite endpoint included ICU admission, which is dependent on provider judgment. Second, the fidelity of our data was limited by the quality of documentation, especially in the evaluation of symptom burden, although standard definitions were used to enhance data quality. Third, multiple confounding factors may influence patient outcomes and were not controlled for in this study. Namely, we did not assess the impact of pre-admission or in-hospital pharmacologic treatments and did not examine the specific complications that developed over the hospital course. In addition, we were unable to evaluate the incubation period of the virus prior to hospitalization, therefore limiting our ability to assess whether duration of infection contributed to disease severity and outcomes. Fourth, although only 12.8% of patients included in the study remained in the hospital at the time of analysis, their ultimate outcomes will alter our final results. Finally, our sample size was small, thus limiting our power to detect less significant differences in patient outcomes. A strength of this study is its use of detailed clinical data points manually abstracted by chart review, as many larger studies relying on coded or structured data are unable to assess the course of disease at this level of granularity. Despite these limitations, this study presents a profile of a regional medical center’s experience in managing hospitalized patients with Covid-19 that may provide useful benchmarks for comparison in similar facilities.

## Conclusion

At our regional medical center, patients with Covid-19 had an average length of stay just under 12 days, required ICU care in 31% of cases, and had a 25% mortality rate. Patients with increased sputum production and higher supplemental oxygen requirements at admission, and insulin dependent diabetes or chronic kidney disease may be at increased risk for severe illness. A model for predicting intensive care unit admission or death with excellent discrimination was created that may aid in treatment decisions and resource allocation. Early identification of patients at increased risk for severe illness may lead to improved outcomes in patients hospitalized with Covid-19.

## Supporting information

S1 Table Variables not included in final regression model. (DOCX)

S2 Table Step summary of model development. (DOCX)

S3 Table Multivariate logistic regression analysis: Presentation predictors of death. (DOCX)

S4 Table Multivariate logistic regression analysis: Presentation predictors of ICU Admission. (DOCX)

S5 Table Multivariate logistic regression analysis: Presentation predictors of ICU admission or death sensitivity analysis with lab values. (DOCX)

S6 Table Cox proportional hazard regression analysis of risk factors and survival to ICU admission or death. (DOCX)
