# Supplementary material for: Risk factors for severe illness in hospitalized Covid-19 patients at a regional hospital
Source: PLoS One. 2020 Aug 12;15(8):e0237558. doi: 10.1371/journal.pone.0237558 (PMC7423129; doi:10.1371/journal.pone.0237558)
Supplement: S1 Table — (DOCX) [file pone.0237558.s001.docx]

**S1 Table.** Variables Not Included in Final Regression Model

| **Variable** | **Score** | **Degrees of Freedom** | | **P-Value** |
| --- | --- | --- | --- | --- |
| Age | .159 | 1 | | .690 |
| Supplemental Oxygen at Admission | 1.050 | 1 | | .306 |
| Myalgia | 1.622 | 1 | | .203 |
| Fatigue | .159 | 1 | | .690 |
| Cough | 1.148 | 1 | | .284 |
| Nausea or Vomiting | 2.606 | 1 | | .106 |
| Obstructive Sleep Apnea | .566 | 1 | | .452 |
| Atrial Fibrillation | 2.311 | 1 | | .128 |
| Coronary Artery Disease | .127 | 1 | | .722 |
| Hypertension | .352 | 1 | | .553 |
| Number of Risk Factors | .087 | 1 | | .768 |
| Number of Symptoms | 1.737 | 1 | | .188 |
| Overall Statistics | 9.331 | | 12 | .674 |
