# Supplementary material for: Risk factors for severe illness in hospitalized Covid-19 patients at a regional hospital
Source: PLoS One. 2020 Aug 12;15(8):e0237558. doi: 10.1371/journal.pone.0237558 (PMC7423129; doi:10.1371/journal.pone.0237558)
Supplement: S2 Table — (DOCX) [file pone.0237558.s002.docx]

**S2 Table.** Step Summary of Model Development

| **Step** | **Improvement** | | | **Model** | | | **Correct Class %** | **Variable** |
| --- | --- | --- | --- | --- | --- | --- | --- | --- |
|  | **Chi-square** | **D.F.** | **P Value** | **Chi-square** | **D.F** | **P Value** |  |  |
| 1 | 16.732 | 1 | <0.001 | 16.732 | 1 | <0.001 | 72.1% | IN: Chronic Kidney Disease |
| 2 | 10.633 | 1 | .001 | 27.365 | 2 | <0.001 | 74.8% | IN: IDDM |
| 3 | 5.919 | 1 | .015 | 33.284 | 3 | <0.001 | 78.4% | IN: Atrial Fibrillation |
| 4 | 5.062 | 1 | .024 | 38.346 | 4 | <0.001 | 73.0% | IN: Amount of Supplemental Oxygen |
| 5 | 4.423 | 1 | .035 | 42.770 | 5 | <0.001 | 77.5% | IN: Sputum |
| 6 | 3.735 | 1 | .053 | 46.505 | 6 | <0.001 | 79.3% | IN: Temperature |
| 7 | -2.287 | 1 | .130 | 44.218 | 5 | <0.001 | 81.1% | OUT: Atrial Fibrillation |
| D.F – degrees of freedom | | | | | | | | |
|  | | | | | | | | |
