# Supplementary material for: Risk factors for severe illness in hospitalized Covid-19 patients at a regional hospital
Source: PLoS One. 2020 Aug 12;15(8):e0237558. doi: 10.1371/journal.pone.0237558 (PMC7423129; doi:10.1371/journal.pone.0237558)
Supplement: S3 Table — (DOCX) [file pone.0237558.s003.docx]

**S3 Table.** Multivariate Logistic Regression Analysis: Presentation Predictors of Death

| **Independent Variable** | **B** |  |  |  | **95% C.I. for Odds Ratio** | |  |
| --- | --- | --- | --- | --- | --- | --- | --- |
|  |  | **S.E.** | **Wald** | **Odds Ratio** | **Lower** | **Upper** | **P Value** |
| Temperature at Admission ( ̊ F ) | -.298 | .135 | 4.846 | 0.743 | 0.570 | 0.968 | **.028** |
| Supplemental O2 at Admission (L/min) | .204 | .085 | 5.837 | 1.227 | 1.039 | 1.448 | **.016** |
| Sputum Production | 1.315 | .807 | 2.658 | 3.726 | 0.766 | 18.112 | .103 |
| Insulin Dependent Diabetes Mellitus | 1.139 | .726 | 2.458 | 3.124 | 0.752 | 12.973 | .117 |
| Chronic Kidney Disease | 2.009 | .610 | 10.844 | 7.454 | 2.255 | 26.640 | .**001** |
| Constant | 26.698 | 13.312 | 4.022 | N/A | N/A | N/A | **.045** |
| Significant P Values < .05 in bold |  |  |  |  |  |  |  |
| O2 - oxygen |  |  |  |  |  |  |  |
