# Supplementary material for: Risk factors for severe illness in hospitalized Covid-19 patients at a regional hospital
Source: PLoS One. 2020 Aug 12;15(8):e0237558. doi: 10.1371/journal.pone.0237558 (PMC7423129; doi:10.1371/journal.pone.0237558)
Supplement: S4 Table — (DOCX) [file pone.0237558.s004.docx]

**S4 Table.** Multivariate Logistic Regression Analysis: Presentation Predictors of ICU Admission

| **Independent Variable** | **B** |  |  |  | **95% C.I. for Odds Ratio** | |  |
| --- | --- | --- | --- | --- | --- | --- | --- |
|  |  | **S.E.** | **Wald** | **Odds Ratio** | **Lower** | **Upper** | **P Value** |
| Temperature at Admission ( ̊ F ) | -.236 | .128 | 3.380 | 0.790 | 0.614 | 1.016 | .066 |
| Supplemental O2 at Admission (L/min) | .056 | .045 | 1.540 | 1.057 | 0.968 | 1.155 | .215 |
| Sputum Production | 1.069 | .689 | 2.409 | 2.913 | 0.755 | 11.239 | .121 |
| Insulin Dependent Diabetes Mellitus | 2.306 | .682 | 11.425 | 10.034 | 2.635 | 38.213 | **.001** |
| Chronic Kidney Disease | -.089 | .601 | .022 | 0.915 | 0.282 | 2.973 | .833 |
| Constant | 21.833 | 12.719 | 2.947 | N/A | N/A | N/A | .086 |
| Significant P Values < .05 in bold |  |  |  |  |  |  |  |
| O2 - oxygen |  |  |  |  |  |  |  |
