# Supplementary material for: Risk factors for severe illness in hospitalized Covid-19 patients at a regional hospital
Source: PLoS One. 2020 Aug 12;15(8):e0237558. doi: 10.1371/journal.pone.0237558 (PMC7423129; doi:10.1371/journal.pone.0237558)
Supplement: S5 Table — (DOCX) [file pone.0237558.s005.docx]

**S5 Table.** Multivariate Logistic Regression Analysis: Presentation Predictors of ICU Admission or Death Sensitivity Analysis with Lab Values

| **Independent Variable** | **B** |  |  |  | **95% C.I. for Odds Ratio** | |  |
| --- | --- | --- | --- | --- | --- | --- | --- |
|  |  | **S.E.** | **Wald** | **Odds Ratio** | **Lower** | **Upper** | **P Value** |
| Temperature at Admission ( ̊ F ) | -.229 | .159 | 2.078 | 0.795 | 0.583 | 1.086 | .149 |
| Supplemental O_2_ at Admission (L/min) | .225 | .144 | 2.437 | 1.252 | 0.944 | 1.660 | .119 |
| Sputum Production | 2.281 | .925 | 6.087 | 9.790 | 1.598 | 59.963 | **.014** |
| Insulin Dependent Diabetes Mellitus | 3.236 | 1.166 | 7.698 | 25.436 | 2.586 | 250.177 | **.006** |
| Chronic Kidney Disease | 1.452 | .697 | 4.345 | 4.272 | 1.091 | 16.736 | **.037** |
| White Blood Cell Count | .052 | .097 | .288 | 1.053 | 0.871 | 1.274 | .591 |
| D-Dimer | .161 | .128 | 1.579 | 1.174 | 0.914 | 1.508 | .209 |
| Constant | 20.229 | 15.714 | 1.657 | N/A | N/A | N/A | .198 |
| Significant P Values < .05 in bold |  |  |  |  |  |  |  |
| S.E. – standard error |  |  |  |  |  |  |  |
| O_2_ - oxygen |  |  |  |  |  |  |  |
