# Supplementary material for: Risk factors for severe illness in hospitalized Covid-19 patients at a regional hospital
Source: PLoS One. 2020 Aug 12;15(8):e0237558. doi: 10.1371/journal.pone.0237558 (PMC7423129; doi:10.1371/journal.pone.0237558)
Supplement: S6 Table — (DOCX) [file pone.0237558.s006.docx]

**S6 Table.** Cox Proportional Hazard Regression Analysis of Risk Factors and Survival to ICU Admission or Death

| **Independent Variable** | **B** |  |  |  | **95% C.I. for Hazard Ratio** | |  |
| --- | --- | --- | --- | --- | --- | --- | --- |
|  |  | **S.E.** | **Wald** | **Hazard Ratio** | **Lower** | **Upper** | **P Value** |
| Temperature at Admission ( ̊ F ) | -.180 | .094 | 3.652 | 0.835 | 0.694 | 1.005 | .056 |
| Supplemental O_2_ at Admission (L/min) | .088 | .026 | 11.061 | 1.092 | 1.037 | 1.150 | **.001** |
| Sputum Production | .793 | .416 | 3.638 | 2.211 | 0.978 | 4.997 | .056 |
| Insulin Dependent Diabetes Mellitus | 1.191 | .373 | 10.194 | 3.292 | 1.584 | 6.840 | **.001** |
| Chronic Kidney Disease | .379 | .362 | 1.096 | 1.461 | 0.718 | 2.973 | .295 |
| Significant P Values < .05 in bold |  |  |  |  |  |  |  |
| S.E. – standard error |  |  |  |  |  |  |  |
| O_2_ - oxygen |  |  |  |  |  |  |  |
